# Antiviral Activity of Influenza A Virus Defective Interfering Particles against SARS-CoV-2 Replication In Vitro through Stimulation of Innate Immunity

**DOI:** 10.3390/cells10071756

**Published:** 2021-07-11

**Authors:** Ulfert Rand, Sascha Young Kupke, Hanna Shkarlet, Marc Dominique Hein, Tatjana Hirsch, Pavel Marichal-Gallardo, Luka Cicin-Sain, Udo Reichl, Dunja Bruder

**Affiliations:** 1 Department of Vaccinology and Applied Microbiology, Helmholtz Centre for Infection Research, 38124 Braunschweig, Germany; Ulfert.Rand@helmholtz-hzi.de (U.R.); Luka.Cicin-Sain@helmholtz-hzi.de (L.C.-S.); 2 Bioprocess Engineering, Max Planck Institute for Dynamics of Complex Technical Systems, 39106 Magdeburg, Germany; marichal-gallardo@mpi-magdeburg.mpg.de (P.M.-G.); ureichl@mpi-magdeburg.mpg.de (U.R.); 3 Immune Regulation Group, Helmholtz Centre for Infection Research, 38124 Braunschweig, Germany; Hanna.Shkarlet@helmholtz-hzi.de (H.S.); Tatjana.Hirsch@helmholtz-hzi.de (T.H.); Dunja.Bruder@helmholtz-hzi.de (D.B.); 4 Institute of Medical Microbiology, Infection Prevention and Control, Infection Immunology Group, Health Campus Immunology, Infectiology and Inflammation, Otto von Guericke University Magdeburg, 39120 Magdeburg, Germany; 5 Bioprocess Engineering, Otto von Guericke University Magdeburg, 39106 Magdeburg, Germany; hein@mpi-magdeburg.mpg.de; 6 German Centre for Infection Research, Hannover-Braunschweig Site, 38124 Braunschweig, Germany; 7 Centre for Individualized Infection Medicine, a Joint Venture of Helmholtz Centre for Infection Research and Medical School Hannover, 38124 Braunschweig, Germany

**Keywords:** SARS-CoV-2, COVID-19, antiviral, influenza A virus, defective interfering particles, OP7, DI244, innate immunity

## Abstract

Severe acute respiratory syndrome coronavirus 2 (SARS-CoV-2) causing coronavirus disease 2019 (COVID-19) emerged in late 2019 and resulted in a devastating pandemic. Although the first approved vaccines were already administered by the end of 2020, worldwide vaccine availability is still limited. Moreover, immune escape variants of the virus are emerging against which the current vaccines may confer only limited protection. Further, existing antivirals and treatment options against COVID-19 show only limited efficacy. Influenza A virus (IAV) defective interfering particles (DIPs) were previously proposed not only for antiviral treatment of the influenza disease but also for pan-specific treatment of interferon (IFN)-sensitive respiratory virus infections. To investigate the applicability of IAV DIPs as an antiviral for the treatment of COVID-19, we conducted in vitro co-infection experiments with cell culture-derived DIPs and the IFN-sensitive SARS-CoV-2 in human lung cells. We show that treatment with IAV DIPs leads to complete abrogation of SARS-CoV-2 replication. Moreover, this inhibitory effect was dependent on janus kinase/signal transducers and activators of transcription (JAK/STAT) signaling. Further, our results suggest boosting of IFN-induced antiviral activity by IAV DIPs as a major contributor in suppressing SARS-CoV-2 replication. Thus, we propose IAV DIPs as an effective antiviral agent for treatment of COVID-19, and potentially also for suppressing the replication of new variants of SARS-CoV-2.

## 1. Introduction

Severe acute respiratory syndrome coronavirus 2 (SARS-CoV-2), causing coronavirus disease 2019 (COVID-19), poses a severe burden to public health, the economy, and society. To date, almost four million deaths are reported in the context of SARS-CoV-2 infection (WHO, covid19.who.int). Since early 2020, there has been an unprecedented race for the development of novel vaccines, their production, and execution of safety and immunogenicity studies in clinical trials [1,2,3,4,5,6]. These efforts led to the vaccination of the first individuals outside clinical trials in late 2020. While vaccination typically provides the best protection against virus infections and disease onset, the worldwide manufacturing capacity of COVID-19 vaccines, and the infrastructure required for vaccination are still limited. In addition, vaccination is a prophylactic measure and not applicable for therapeutic treatment of acute infections. Therefore, as an alternative option, the development of antivirals for treatment of COVID-19 is essential. Yet, remdesivir (in clinical use) showed only limited efficacy [7,8,9], while other repurposed drug candidates (e.g., hydroxychloroquine and lopinavir-ritonavir) showed a lack of efficacy [10,11]. In addition, corticosteroids (i.e., dexamethasone [12]) and cocktails of monoclonal antibodies (e.g., bamlanivimab [13]) are used in the clinic and show an antiviral effect. However, the appearance of new SARS-CoV-2 variants poses a constant risk of losing efficacy of highly specific treatments, including vaccination and therapeutic antibodies. Thus, there is a need to develop more broadly acting, cost-effective antivirals that ideally are easily scalable in production.

Influenza A virus (IAV) defective interfering particles (DIPs) were previously proposed for antiviral treatment against IAV infections [14,15,16,17,18,19,20,21,22], but also for pan-specific treatments of other respiratory viral diseases [23,24]. IAV DIPs typically carry a large internal deletion in their genome, rendering them defective in virus replication [25,26,27]. Furthermore, DIPs suppress and interfere specifically with homologous viral replication in a co-infection scenario, a process known as replication interference. As a result, administration of IAV DIPs in mice resulted in full protection against an otherwise lethal IAV infection [21,28,29,30]. In the ferret model, treatment of IAV-infected animals resulted in a reduced severity of disease pathogenesis [31]. Mice were also protected against a lethal infection with the unrelated influenza B virus [32] and pneumonia virus of mice (PVM) from the family *Paramyxoviridae* [33]. Here, protection was not attributed to replication interference but to the ability of IAV DIPs to stimulate innate immunity.

SARS-CoV-2 replication seems to modulate and inhibit the interferon (IFN) response in infected target cells [34,35,36]. Still, it was shown to be susceptible to inhibition by exogenously added IFNs in vitro [37,38], in vivo [39], and in clinical trials [40]. Therapies using recombinant IFNs, however, are cost intensive and pose the risk of unwanted side effects including the formation of auto-antibodies against cytokines (reviewed in [41]). To prevent this, we speculate whether IAV DIPs can suppress SARS-CoV-2 replication through their ability to stimulate a physiological IFN response in target cells. To test this, we produced two promising candidate DIPs: a prototypic, well-characterized conventional IAV DIP “DI244” [23] and a novel type of IAV DIP “OP7” that contains point mutations instead of a large internal deletion in the genome [42], using a cell culture-based production process [29,30,43].

Here, we used Calu-3 cells (human lung cancer cell line) for in vitro co-infection experiments with SARS-CoV-2 and DI244 or OP7. Both DIPs were able to completely inhibit SARS-CoV-2 replication and spreading in a range comparable to IFN-β or remdesivir treatment. Moreover, we show that the inhibitory effect of IAV DIPs was due to their ability to induce innate immune responses that signal via janus kinase/signal transducers and activators of transcription (JAK/STAT). In addition, our results show that IAV DIP infection triggers elevated host cell type-I and type-III IFN production and subsequent IFN-induced antiviral activity. Thus, we propose IAV DIPs as effective antiviral agents for the treatment of COVID-19 and, potentially as universal antiviral agents not only against different influenza subtypes but also against other (including newly emerging) IFN-sensitive respiratory viruses.

## 2. Materials and Methods

### 2.1. Cells and Viruses

Vero-6 cells (ATCC (Manassas, VA, USA), #CRL-1586) were maintained in DMEM medium (Gibco (Carlsbad, CA, USA), 4.5 g/L glucose, w/o pyruvate), supplemented with 10% fetal calf serum (FCS, Biowest (Nuaillé, France), #S1810-6500), 100 IU/mL penicillin, 100 μg/mL streptomycin, 1× GlutaMax (Gibco), and 1× sodium pyruvate (Gibco). Calu-3 cells (ATCC, #HTB-55) were cultured in MEM (Sigma, St. Louis, MO, USA) supplemented with 10% FCS (Biowest, #S1810-6500), 100 IU/mL penicillin, 100 μg/mL streptomycin, 1× GlutaMax (Gibco), and 1× sodium pyruvate (Gibco). Caco-2 cells (ATCC, #HTB-37) were grown in MEM (Gibco), supplemented with 20% FCS (Biowest, #S1810-6500), 100 IU/mL penicillin, 100 μg/mL streptomycin, 1× GlutaMax (Gibco), and 1× non-essential amino acid solution (Gibco). All cells were maintained or infected at 37 °C in a 5% CO_2_ atmosphere.

The IAV DIPs DI244 and OP7 were produced in a cell culture-based process using a 500 mL laboratory scale stirred tank bioreactor, followed by purification and concentration by membrane-based steric exclusion chromatography [44,45], as described previously [29,30]. Production titers of 3.3 and 3.67 log hemagglutination (HA) units/100µL (quantified by the HA assay [46]) and 5.6 × 10^8^ and 1.12 × 10^11^ DI vRNAs/mL (quantified by real-time RT-qPCR [29,42,47]) were achieved for DI244 and OP7, respectively.

The SARS-CoV-2 isolate hCoV-19/Croatia/ZG-297-20/2020 was used. All experiments with infectious SARS-CoV-2 were performed in the BSL-3 facility at the Helmholtz Centre for Infection Research (Braunschweig, Germany). The SARS-CoV-2 seed virus was produced in Caco-2 cells, and virus particles were enriched in Vivaspin 20 columns (Sartorius, Göttingen, Germany) via centrifugation. Collected virus was stored at −80 °C. SARS-CoV-2 titers were quantified by plaque assay.

### 2.2. SARS-CoV-2 Quantification

Quantification of SARS-CoV-2 was performed by plaque assay. Samples were serially diluted in 10-fold steps and used to infect a confluent monolayer of Vero-6 cells (on 96-well plates) for 1 h. Then, the inoculum was removed, and cells were overlaid with cell culture medium containing 1.5% methyl-cellulose (Sigma, #C9481-500). At 3 dpi, cells were fixed with 6% formaldehyde and stained with crystal violet. Wells were imaged using a Sartorius IncuCyte S3 (4× objective, whole-well scan) and plaque counts were determined.

### 2.3. SARS-CoV-2 Infection and Antiviral Treatment

Confluent Calu-3 cells in 96-well plates (~6 × 10^4^ cells/well) were infected with SARS-CoV-2 (2000 pfu per well). At 1 or 24 hpi, we added active or inactive IAV DIPs (DI244 or OP7) at indicated fractions (% *v*/*v*) with respect to the cell culture volume of 100 µL. Whenever indicated, we additionally added 0.8 µM ruxolitinib (Cayman Chemical (Ann Arbor, MI, USA), #Cay11609-1) to these wells. Alternatively, remdesivir (MedChem Express (Monmouth Junction, NJ, USAnited States), #HY-104077) or human IFN-β-1A (PBL assay science (Piscataway, NJ, USA), #11415-1) (instead of IAV DIPs) were added at indicated concentrations at 1 hpi. Supernatants were collected at indicated time points for quantification of SARS-CoV-2 titers (plaque assay) and for protein quantification of secreted IFNs using commercially available ELISA kits (see below). In addition, infected cells were lysed using solution RL for subsequent total RNA extraction using the innuPREP RNA Mini Kit 2.0 (Analytik Jena (Jena, Germany), #845-KS-2040050), according to the manufacturer’s instructions, for gene expression analysis via real-time RT-qPCR.

### 2.4. Immunofluorescence Staining

(Co-)infected cells were fixed with 6% paraformaldehyde in PBS for 1 h at room temperature, followed by washing with PBS. Cells were permeabilized with 0.1% Triton X-100 in PBS for 10 min at room temperature, washed with PBS, and blocked with 2% BSA in PBS for 1 h. Antibody labelling was performed with mouse anti-SARS-CoV-2 S protein (Abcalis (Braunschweig, Germany), #ABK68-A09-M) and secondary antibody anti-mouse Alexa488 (Cell Signaling Technology (Danvers, MA, USA), #4408), each step was followed by three washing steps with PBS containing 0.05% Tween-20. Finally, cells were overlaid with Vectashield Mounting Medium (Biozol (Eching, Germany), #VEC-H-1000).

### 2.5. Assessment of IFN Production by Host Cells

Supernatants of (co-)infected cells were assessed for IFN-β and IFN-λ3 levels using corresponding Quantikine ELISA kits (R&D Systems (Minneapolis, MN, USA), #DIFNB0 and #D28B00, respectively) according to the manufacturer’s instructions.

### 2.6. Gene Expression Analysis

mRNA expression levels in (co-)infected cells were assessed using real-time RT-qPCR. 500 ng of total RNA were reverse transcribed using an oligo(dT) primer and the enzyme Maxima H Minus (both from Thermo Scientific, Waltham, MA, USA) according to the manufacturer’s instructions. Next, qPCR was conducted using the Rotor-Gene Q real-time PCR cycler (Qiagen, Hilden, Germany) and the following primers: *IFNB1*, 5’-CATTACCTGAAGGCCAAGGA-3′ and 5′-CAGCATCTGCTGGTTGAAGA-3′; *IFNL1*, 5′-GGTGACTTTGGTGCTAGGCT-3′ and 5′-TGAGTGACTCTTCCAAGGCG-3′; *MX1*, 5′-GTATCACAGAGCTGTTCTCCTG-3′ and 5′-CTCCCACTCCCTGAAATCTG-3′; *RSAD2*, 5′-CCCCAACCAGCGTCAACTAT-3′ and 5′-TGATCTTCTCCATACCAGCTTCC-3′; *GAPDH*, 5′-CTGGCGTCTTCACCACCATGG-3′ and 5′-CATCACGCCACAGTTTCCCGG-3′ (all sequences from [37]); and *DDX58*, 5′-TGCAAGCTGTGTGCTTCTCT-3′ and 5′-TCCTGAAAAACTTCTGGGGCT-3′ [48]. The qPCR reaction mixture (10 μL) comprised 1× Rotor-Gene SYBR green PCR mix (Qiagen), 500 nM of each primer, and 4 μL of cDNA. DNA denaturation was conducted for 5 min at 95 °C, followed by 40 PCR cycles: 10 s at 95 °C and 20 s at 62 °C. Gene expression was calculated using the ΔΔCT method using *GAPDH* as the reference gene and expressed as fold change relative to untreated, uninfected cells.

### 2.7. Quantification of Intracellular IAV DI vRNAs

Real-time RT-qPCR was used for quantification of intracellular DI vRNAs from purified total RNA, as described previously [29,30,42,47]. In brief, a primer system was used that allows polarity- and gene-specific detection of individual IAV vRNAs [49]. To enable absolute quantification, RNA reference standards were synthesized and levels of vRNAs were calculated based on standard curves, as described previously [42].

For RT, 1 µL of the total RNA sample was combined with 1 µL of dNTPs (10 mM) and 1 µL of the RT primer (1 µM) and filled up to a volume of 15 µL with nuclease-free water. Incubation was performed at 65 °C for 5 min and 55 °C for 5 min. During the latter step, a prewarmed mixture (55 °C) consisting of 4 µL of 5x RT buffer, 0.5 µL (100 U) Maxima H Minus reverse transcriptase, and 0.5 µL RiboLock RNase Inhibitor (all reagents from Thermo Scientific) was added. RT primer: OP7, 5′-ATTTAGGTGACACTATAGAAGCGACTGTGACTGCTGAAGTGGTG-3′; DI244, 5′-ATTTAGGTGACACTATAGAAGCGAGCGAAAGCAGGTCAATTATATTC-3′. RT was conducted for 30 min at 60 °C, followed by 85 °C for 5 min. Further, RNA reference standards in 10-fold dilution steps were reverse transcribed. Next, the cDNA reaction products were diluted to 100 µL in nuclease-free water. For qPCR, the Rotor-Gene Q real-time PCR cycler (Qiagen) was used. The qPCR mix (10 µL) contained 1× Rotor-Gene SYBR green PCR mix (Qiagen), 500 nM of each primer, and 4 µL of diluted cDNA. qPCR Primers: OP7, 5′-ATTTAGGTGACACTATAGAAGCG-3′ and 5′-CATTTGCCTAGCCCGAATC-3′; DI244, 5′- ATTTAGGTGACACTATAGAAGCG-3′ and 5′-GGAATCCCCTCAGTCTTC-3′. The PCR cycling conditions comprised: initial denaturation step at 95 °C for 5 min, followed by 40 PCR cycles of 95 °C for 10 s and 62 °C for 20 s.

### 2.8. Data Sharing

**Figure 1:**
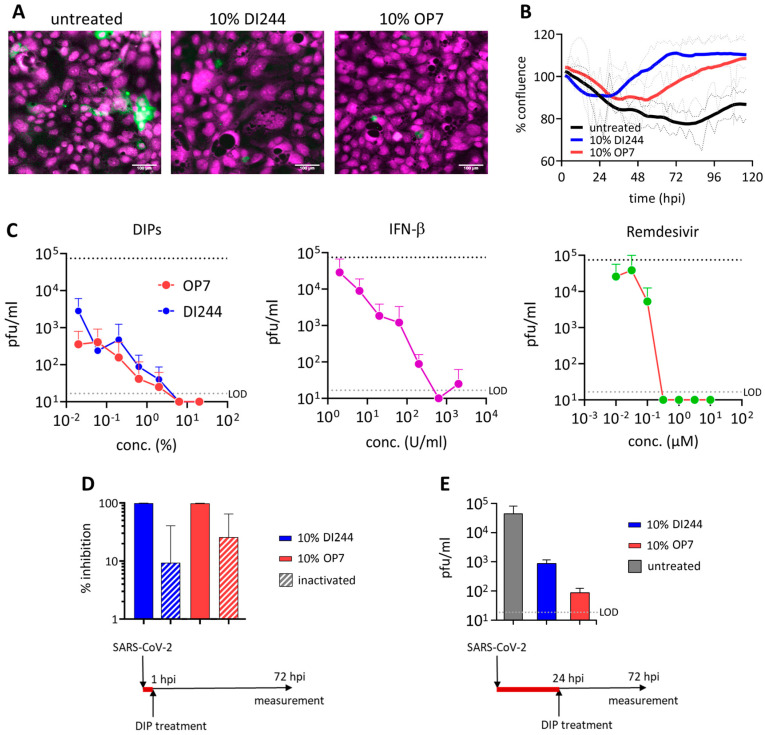
Inhibition of severe acute respiratory syndrome coronavirus 2 (SARS-CoV-2) replication and spreading by influenza A virus (IAV) defective interfering particles (DIPs). SARS-CoV-2-infected Calu-3 cells (multiplicity of infection (MOI) = 0.03) were treated with IAV DIPs (DI244 or OP7), interferon (IFN)-β, or remdesivir at 1 hour post infection (hpi). For DI244 and OP7 treatment, chromatographically purified and highly concentrated cell culture-derived DIP material [29,30] was used. % (*v*/*v*) indicates the fraction with respect to the cell culture volume of 100 µL. Stock concentration, 5.6 × 10^8^ and 1.12 × 10^11^ defective interfering (DI) viral RNAs (vRNAs)/mL for DI244 and OP7, respectively. (**A**) Immunofluorescence analysis of the SARS-CoV-2 spike (S) protein expression (green, magenta: DNA) at 3 dpi. Scale bar, 100 µm. (**B**) Cytopathic effect. Confluence (% of initial) was measured by live-cell microscopy at 2 h intervals. Thick lines represent smoothened data (Savitzky-Golay filter), dotted lines show SD of original data (*n* = 2, independent experiments). (**C**) Effective concentration range of DI244 and OP7 compared to IFN-β and remdesivir. Viral titers were determined from the supernatant at 3 days post infection (dpi) by plaque assay. Upper dotted line indicates virus titer in untreated cells, lower dotted line shows the limit of detection (LOD). Independent experiments were conducted; mean ± SD (*n* = 3) is shown. pfu, plaque-forming units. (**D**) SARS-CoV-2 growth inhibition by active and inactive DIPs. SARS-CoV-2 infected cells were treated with active or ultraviolet (UV)-irradiated (inactivated) DIPs at 1 hpi. Percentage inhibition of viral growth relative to mock treatment is shown; mean ± SEM (*n* = 4) is depicted. (**E**) DIP superinfection 24 h post SARS-CoV-2 infection. Independent experiments were conducted; mean ± SD (*n* = 2) is shown.

**Figure 2:**
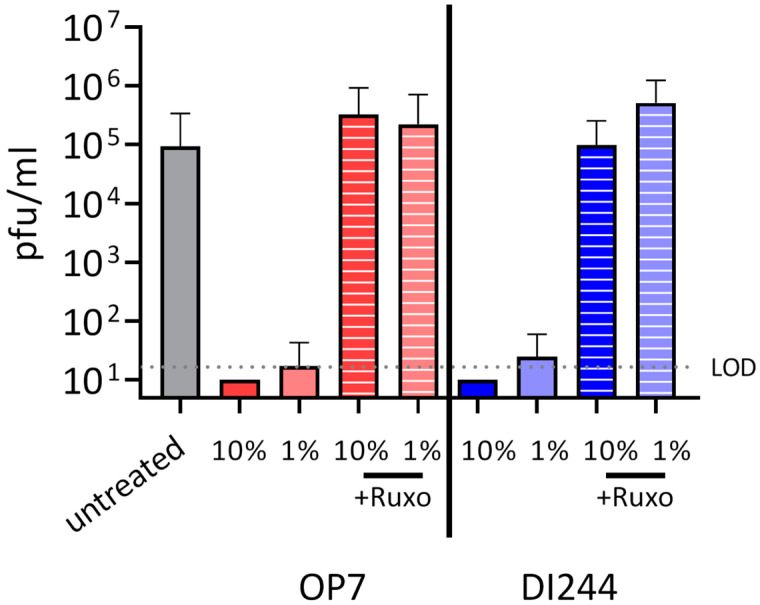
Suppression of SARS-CoV-2 replication by IAV DIPs under janus kinase (JAK) inhibition. SARS-CoV-2-infected Calu-3 cells (MOI = 0.03) were co-infected with IAV DIPs (DI244 or OP7) at 1 hpi in the presence or absence of ruxolitinib (JAK inhibitor). % (*v*/*v*) indicates the fraction of DIPs (highly concentrated cell culture-derived material) [29,30] with respect to the cell culture volume of 100 µL. Viral titers were determined from the supernatant at 3 dpi by plaque assay. Dotted line shows the LOD. Independent experiments were conducted; mean ± SD (*n* = 4) is depicted.

**Figure 3:**
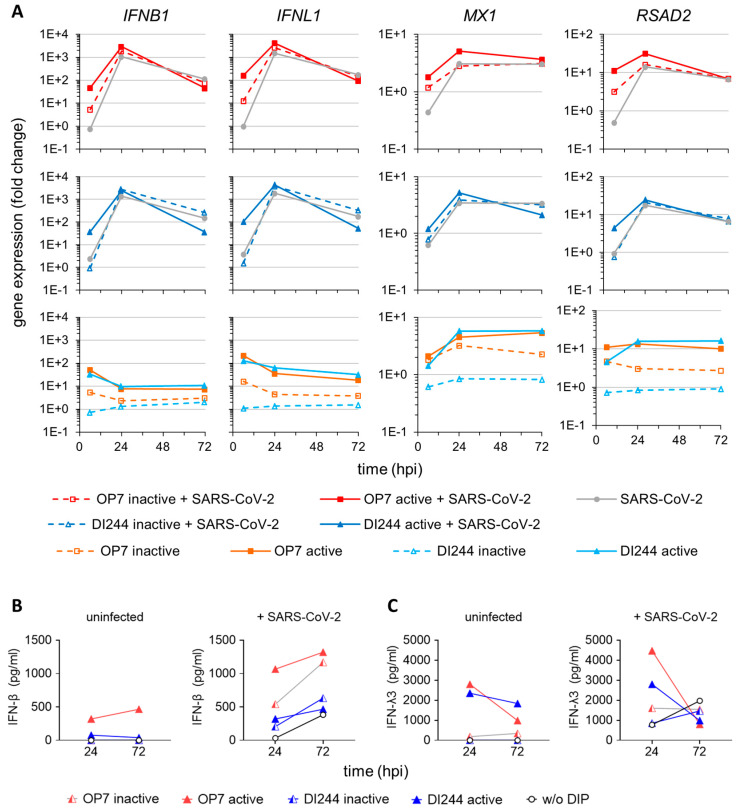
Stimulation of IFN-induced antiviral activity by IAV DIP infection. SARS-CoV-2-infected Calu-3 cells (MOI = 0.03) were treated with IAV DIPs (DI244 or OP7) at 1 hpi. For DI244 and OP7 infection, 10% (*v*/*v*) (100 µL culture volume) of highly concentrated cell culture-derived DIP material was used [29,30]. At indicated times post-infection, infected cells were lysed to allow for total RNA extraction, required for (**A**) gene expression analysis. In addition, supernatants were sampled for (**B**,**C**) quantification of secreted IFNs. Illustration includes data from one experiment. (**A**) Gene expression analysis of SARS-CoV-2 and IAV DIP co-infection. Transcript levels were quantified by real-time RT-qPCR and expressed as fold change (relative to untreated, uninfected cells). *MX1*, *MX dynamin-like GTPase 1*; *RSAD2*, *radical S-adenosyl methionine domain-containing 2*. (**B**,**C**) Host cell IFN production. Protein levels of IFN-β (**B**) and IFN-λ3 (**C**) were assessed using ELISA.

**Figure 4:**
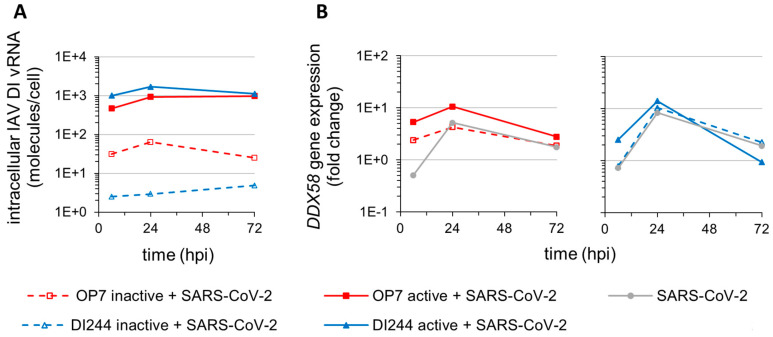
Cellular uptake of IAV DI vRNAs and *DExD/H-box helicase 58* (*DDX58*) expression. SARS-CoV-2-infected Calu-3 cells (MOI = 0.03) were treated with IAV DIPs (DI244 or OP7) at 1 hpi. For DI244 and OP7 infection, 10% (*v*/*v*) (100 µL culture volume) of highly concentrated cell culture-derived DIP material was used [29,30]. At indicated times post-infection, cells were lysed to allow for total RNA extraction, required for (**A**) quantification of intracellular DI vRNAs and (**B**) analysis of *DDX58* gene expression. Illustration includes data from one experiment. (**A**) Intracellular DI vRNA levels during SARS-CoV-2 and IAV DIP co-infection. Cells were assayed for viral RNAs by real-time RT-qPCR. (**B**) *DDX58* gene expression analysis. *DDX58* (encoding for RIG-I) transcript levels were quantified by real-time RT-qPCR and expressed as fold change (relative to untreated, uninfected cells).

The full dataset (of Figures 1–4) is available in supplemental Table S1.

## 3. Results

### 3.1. SARS-CoV-2 Replication Is Abrogated by IAV DIP Treatment In Vitro

In order to test the antiviral efficacy of IAV DIPs on replication of SARS-CoV-2, we conducted in vitro co-infection experiments in Calu-3 cells. For this, we infected cells with SARS-CoV-2 (multiplicity of infection (MOI) = 0.03) and highly concentrated IAV DIPs (DI244 or OP7) from cell culture-based production and membrane-based chromatographic purification [29,30,44]. At 3 days post infection (dpi), cells were stained for the SARS-CoV-2 spike (S) protein (Figure 1A). Indeed, S protein expression was significantly reduced in cells co-treated with DI244 or OP7 compared with cells infected with SARS-CoV-2 only, indicating suppression of SARS-CoV-2 replication by IAV DIP co-infection. In agreement with this observation, images from live-cell microscopy showed a clearly reduced cytopathic effect upon DIP co-infection from ~36 hours post infection (hpi) (Figure 1B).

To test the antiviral efficiency of IAV DIPs in comparison with clinically relevant antivirals, dose effect curves were generated by testing different concentrations of IAV DIPs for the treatment of SARS-CoV-2-infected cells (Figure 1C). As a read-out for SARS-CoV-2 replication, we determined the infectious titer from supernatants at 3 dpi using plaque assays with Vero-6 cells. Infection with only defective, replication-incompetent IAV DIPs does not result in the release of progeny virions, as demonstrated by negative plaque titers [29,30]. Strikingly, SARS-CoV-2 replication was severely diminished upon IAV DIP co-infection. In particular, at high DI244 and OP7 concentrations, SARS-CoV-2 plaque titers were no longer detectable, while untreated cells showed a titer of 7.5 × 10^4^ plaque-forming units (pfu)/mL. Suppression of SARS-CoV-2 replication decreased with increasing dilution of DIPs. Remarkably, though, the treatment with both DIPs at a dilution of 1:5000 still resulted in a pronounced inhibition of SARS-CoV-2 replication, corresponding to a 26-fold and 210-fold reduction in plaque titers for DI244 and OP7 treatment, respectively. OP7 showed an overall slightly higher interfering efficacy than DI244 (Figure 1), in agreement with recent viral challenge studies involving IAV, conducted in vitro and in vivo [29,30]. For comparison, we also tested the inhibitory capacity of IFN-β, or remdesivir treatment, on SARS-CoV-2 replication in infected target cells. Both agents were also able to diminish SARS-CoV-2 plaque titers to below the limit of detection (LOD), until a concentration of 633 U/mL for IFN-β and 0.32 µM for remdesivir. Yet, inhibiting effects ceased significantly faster with increasing dilutions, most apparently observed for remdesivir, for which treatment with a concentration of 0.03 µM no longer resulted in an inhibitory effect.

Figure 1D illustrates residual SARS-CoV-2 inhibition caused by inactivated DIPs. These DIPs were previously ultraviolet (UV)-irradiated until no interfering efficacy against IAV replication was observed in vitro [29,30], indicating the degradation of the causative interfering agent, i.e., the genomic defective interfering (DI) viral RNA (vRNA). The finding that inhibition caused by active DIPs was more efficient suggests a specific interfering activity of active IAV DIPs with SARS-CoV-2 replication. Of note, active DIPs still conferred a pronounced antiviral effect even when applied 24 h after preceding SARS-CoV-2 infection (Figure 1E).

In conclusion, treatment with both DI244 and OP7 IAV DIPs completely inhibited SARS-CoV-2 replication during in vitro co-infections. While the inhibitory potential was comparable to IFN-β and remdesivir treatment, the antiviral effects of IAV DIPs were more sustained with increasing dilution.

### 3.2. IAV DIP Infection Enhances Type-I and Type-III IFN Responses and Inhibit SARS-CoV-2 Replication via Janus Kinase Signaling

Next, to investigate our hypothesis of whether inhibition of SARS-CoV-2 replication by DIPs is due to their ability to stimulate the IFN system, we used ruxolitinib in co-infection experiments. This small molecule drug is an efficient inhibitor of JAK, which are key effectors in the IFN system. Upon IFN sensing, JAKs typically recruit STATs, ultimately leading to the upregulation of IFN-stimulated genes (ISGs). ISGs encode for effector molecules that limit viral replication by inducing an antiviral state in the infected as well as uninfected neighboring cells. Figure 2 shows the results of SARS-CoV-2 and IAV DIP co-infection upon treatment with ruxolitinib. While DI244 and OP7 co-infection almost completely inhibited SARS-CoV-2 replication, additional treatment with ruxolitinib abrogated the suppressive effect of both IAV DIPs. Specifically, virus titers under JAK signaling inhibition were comparable to SARS-CoV-2 infection in the absence of DIPs. These results suggest a major contribution of unspecific innate immune activation by IAV DIPs in interfering with SARS-CoV-2 replication.

In order to investigate correlates of IAV DIP-mediated SARS-CoV-2 suppression, we used real-time RT-qPCR for quantification of gene expression to assess innate immune responses during co-infections in more detail (Figure 3A). We observed a significant upregulation of type I and III IFN (i.e., *IFNB1* and *IFNL1*, respectively) expression at early times following IAV DIP and SARS-CoV-2 co-infection compared with SARS-CoV-2 single infection. Specifically, we detected one to two log_10_ higher *IFNB1* and *IFNL1* mRNA levels at 6 hpi. In addition, canonical antiviral gene expression, indicated by *MX dynamin-like GTPase 1* (*MX1*) and *radical S-adenosyl methionine domain-containing 2* (*RSAD2*), was elevated. This early upregulation of innate immune responses may well explain the strong antiviral activity of DI244 and OP7 against SARS-CoV-2 replication.

In agreement with this, we detected significant uptake of the nonreplicating DI244 and OP7 vRNA (~10^3^ molecules/cell) (Figure 4A) and concomitant upregulation of *DExD/H-box helicase 58* (*DDX58*) (Figure 4B), encoding for retinoic acid inducible gene I (RIG-I); a pattern recognition receptor (PRR) critical for detection of viral nucleic acids and initiation of cellular antiviral responses. The significantly lower levels of DI vRNAs detected for cells treated with inactive DIPs can be explained by their efficient degradation by the inactivation procedure (i.e., UV-irradiation). In this context, the trend toward stronger stimulation of IFN-induced antiviral activity by inactive OP7 compared with inactive DI244 (Figure 3) may be well explained by the higher DI vRNA levels detected upon inactive OP7 treatment (Figure 4A).

As a control, we infected cells with only active or inactive IAV DIPs. Here, only active DIPs were able to mount a marked innate immune response (Figure 3A, lower panels). Canonical antiviral gene expression (*MX1* and *RSAD2*) was enhanced until 72 hpi for the active DIP-only treatment, implying that DI244 and OP7 can potentially raise a long-lived antiviral ISG response.

Consistent with the results from real-time RT-qPCR, protein levels of secreted IFN-β and IFN-λ3 (investigated using ELISA) were elevated at later times post-infection for cells co-infected with IAV DIPs and SARS-CoV-2 compared with cells infected with only SARS-CoV-2 (Figure 3B and 3C). In conclusion, our results suggest the early stimulation and subsequent boosting of the type-I and type-III IFN response as causative for the inhibiting potential of IAV DIPs against SARS-CoV-2 replication.

## 4. Discussion

Despite the recent availability of vaccines against COVID-19, options for antiviral treatment are urgently needed for therapeutic application. Here, we show that cell culture-derived IAV DIPs are highly potent inhibitors of SARS-CoV-2 replication in human lung cells. In addition, our data obtained from in vitro experiments suggest that suppression of SARS-CoV-2 replication by IAV DIPs is predominantly attributed to their ability to stimulate innate immune responses ultimately inducing an antiviral state in target cells.

In the clinic, already approved antivirals for treatment of COVID-19 showed only limited efficacy. For instance, treatment with the polymerase inhibitor remdesivir did not result in an overall decrease in mortality [8,9]. For patients receiving supplemental oxygen, however, an improvement in the survival rate from 4% to 12% was observed [8]. In addition, the time required to recover from COVID-19 was decreased by five days [7,8]. Another option to treat COVID-19 is the use of monoclonal antibodies that target the receptor binding domain of the SARS-CoV-2 S protein, thereby inhibiting engagement with the host cell entry receptor angiotensin-converting enzyme 2 (ACE2) [50,51]. Here, it was suggested to use antibody cocktails to prevent the emergence of viral escape variants in treated individuals [52]. In clinical trials, treatment of outpatients with one such antibody cocktail (i.e., bamlanivimab) accelerated the decrease in viral load and reduced the fraction of patients requiring hospitalization from 6.3% to 1.6% [13]. The administration of the corticosteroid dexamethasone (in clinical use) resulted in an overall lower mortality in critically ill COVID-19 patients [53,54]. This has a caveat, though, as a decrease in mortality was observed for patients requiring oxygen (including mechanical ventilation), but an increase in mortality was reported for patients not requiring oxygen [53]. This comparatively little progress in COVID-19 therapy has sparked calls to invest more research into broadly acting antiviral agents against SARS-CoV-2 to combat future pandemics [55].

Treatment of COVID-19 patients with IFNs is not approved yet. In general, SARS-CoV-2 infection can modulate and inhibit the IFN response [34,35,36]. In addition, it was recently shown that the host cell entry receptor ACE2 is indeed an ISG, and it was speculated that SARS-CoV-2 may exploit the IFN-driven upregulation of ACE2 to enhance infection [56]. However, SARS-CoV-2 replication is, in general, also susceptible to inhibition by exogenously added IFN. For instance, all IFNs (type I, II, and III) exhibited potent antiviral activity with SARS-CoV-2 replication in vitro [37,38], suggesting that the antiviral activities of IFNs can counterbalance any pro-viral effects derived from ACE2 induction. Yet, type-I IFN treatment seems to play an ambiguous role in COVID-19, depending on the stage of the disease (reviewed in [57]), and also type-III IFNs can contribute to COVID-19 pathogenesis [58]. Therefore, therapeutic administration of IFNs (or IAV DIPs) in the future will have to be carefully evaluated with respect to timing. Nevertheless, it was shown that intranasal IFN-I administration (in hamsters) pre- or post-virus challenge can reduce SARS-CoV-2 disease burden [39]. Moreover, in a placebo-controlled phase 2 clinical trial, administration of inhaled, nebulized IFN-β (to patients already admitted to the hospital due to COVID-19 symptoms) resulted in a higher chance of disease improvement and a more rapid recovery from COVID-19 [40].

In our cell culture experiments, IAV DIPs completely abrogated SARS-CoV-2 replication. Notably, the UV-irradiated and thus inactive DIP material (containing degraded DI vRNAs) also showed a residual inhibitory effect. Yet, the observation of a much stronger antiviral effect upon treatment with active DIPs hints to an immunostimulatory activity of active IAV DIPs in the context of SARS-CoV-2 suppression. In principle, DIPs are defective in virus replication; therefore, they fail to complete the entire infection cycle. Thus, the genomic DI vRNAs entering the cell cannot multiply, but are typically very well detected by RIG-I [59], which subsequently leads to the activation of an IFN-response [60], in line with our results. In conclusion, it appears that physically (or chemically) inactivated viral particles are not sufficient to induce a strong antiviral immunity, in contrast to “live” but propagation-incompetent IAV DIPs. Furthermore, infection with rhinovirus before SARS-CoV-2 in vitro can also result in an accelerated ISG responses and a prevention of SARS-CoV-2 replication [61]; yet, fully infectious and propagation-competent rhinovirus was used, which may result in unfavorable side effects. With respect to the safety of IAV DIPs for potential clinical application, it is important to note that administration of only active DIPs did not cause apparent toxic effects in mice and ferrets [28,29,30,31].

Our results support the notion that IAV DIPs do not only protect host cells from IAV infection but, in addition, may generally confer protection against other heterologous IFN-sensitive respiratory viruses [24,32,33]. Considering the emergence of new SARS-CoV-2 variants, against which a decreasing efficacy of various vaccines is becoming evident, the unspecific stimulation of innate immunity by IAV DIPs is considered advantageous; in particular, regarding a potential universal efficacy against such new (and future) variants. Furthermore, previous in vitro and in vivo experiments also revealed an antiviral effect of IAV DIPs against a variety of different IAV subtypes, including pandemic and highly pathogenic avian IAV strains [14,28,31,62].

Future work to clarify the therapeutic effects of IAV DIPs on the outcome of SARS-CoV-2 infection and to decipher in more detail the underlying mode of action should comprise animal trials in Syrian hamsters or genetically modified humanized K18-hACE2 mice, which are susceptible to SARS-CoV-2 infection and develop a similar respiratory disease compared with human COVID-19 [63,64,65,66]. Animal experiments will help to elaborate on the potential applicability of IAV DIPs (e.g., administration via a droplet spray) as a pre- and post-exposure treatment for instance in acute SARS-CoV-2 outbreak scenarios in clinics or geriatric institutions. In addition to vaccination, this represents an interesting option for prophylactic treatment to boost antiviral immunity in persons at acute risk for an infection or for therapeutic treatment during early phase post-infection and, as such, may prevent fatal COVID-19 outcomes.

## 5. Patents

A patent for the use of OP7 as an antiviral agent for treatment of IAV infection is pending. Patent holders are S.Y.K. and U.R. (Udo Reichl).

Another patent for the use of DI244 and OP7 as an antiviral agent for treatment of coronavirus infection is pending. Patent holders are S.Y.K., U.R. (Udo Reichl), M.H., U.R. (Ulfert Rand), and D.B.

P.M.G. and U.R. (Udo Reichl) are inventors in a pending patent application detailing the technology used for the chromatographic purification of the influenza virus particles used in this study.

## Data Availability

Full dataset (of Figure 1, Figure 2, Figure 3 and Figure 4) is available in supplemental Table S1.

## Supplementary Materials

The following are available online at https://www.mdpi.com/article/10.3390/cells10071756/s1, Table S1: Full dataset of Figure 1, Figure 2, Figure 3 and Figure 4.
